# Current Progress in Vascular Engineering and Its Clinical Applications

**DOI:** 10.3390/cells11030493

**Published:** 2022-01-31

**Authors:** Hatem Jouda, Luis Larrea Murillo, Tao Wang

**Affiliations:** 1 Manchester Medical School, Faculty of Biology, Medicine and Health, University of Manchester, Manchester M13 9PL, UK; hatem.jouda@student.manchester.ac.uk; 2 School of Biological Sciences, Faculty of Biology, Medicine and Health, University of Manchester, Manchester M13 9PL, UK; luis.larrea@postgrad.manchester.ac.uk

**Keywords:** vascular tissue engineering, ischemic heart disease, tissue engineered vascular grafts, induced pluripotent stem cells, mesenchyme stem cells

## Abstract

Coronary heart disease (CHD) is caused by narrowing or blockage of coronary arteries due to atherosclerosis. Coronary artery bypass grafting (CABG) is widely used for the treatment of severe CHD cases. Although autologous vessels are a preferred choice, healthy autologous vessels are not always available; hence there is a demand for tissue engineered vascular grafts (TEVGs) to be used as alternatives. However, producing clinical grade implantable TEVGs that could healthily survive in the host with long-term patency is still a great challenge. There are additional difficulties in producing small diameter (<6 mm) vascular conduits. As a result, there have not been TEVGs that are commercially available. Properties of vascular scaffolds such as tensile strength, thrombogenicity and immunogenicity are key factors that determine the biocompatibility of TEVGs. The source of vascular cells employed to produce TEVGs is a limiting factor for large-scale productions. Advanced technologies including the combined use of natural and biodegradable synthetic materials for scaffolds in conjunction with the use of mesenchyme stem cells or induced pluripotent stem cells (iPSCs) provide promising solutions for vascular tissue engineering. The aim of this review is to provide an update on various aspects in this field and the current status of TEVG clinical applications.

## 1. Background

**Figure 1:**
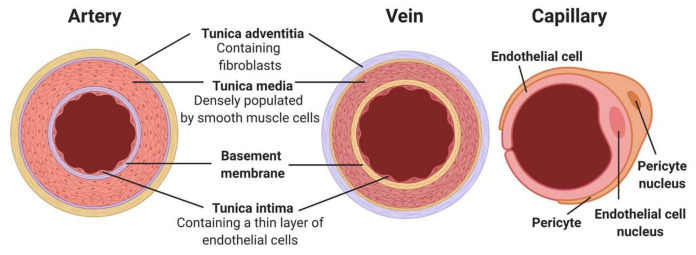
Structure of blood vessels. Diagram shows compositions of the three main types. From left to right: artery, vein and capillary. (Created with BioRender.com, accessed on 8 January 2022).

Healthy blood vessels are integral to body function. They provide tissues with nutrients and oxygen, as well as remove waste products such as carbon dioxide and metabolites. With the exception of capillaries, all blood vessels are composed of three main cellular layers: the tunica intima, tunica media and tunica adventitia (Figure 1) [1,2]. The major cell types that compose these layers are endothelial cells (ECs) for the intima, vascular smooth muscle cells (VSMCs) for the media and fibroblasts for the adventitia. There are structural differences among different types of vessels, e.g., arteries versus veins, small resistance arteries versus large conduit arteries, in addition to different compositions of the extracellular matrix (ECM), which support and regulate specific functions of blood vessels [3,4]. Certain risk factors induce pathological changes in blood vessels, leading to common cardiovascular conditions. For example, endothelial damages caused by smoking, obesity, and aging lead to atherosclerosis, which can ultimately manifest into coronary heart disease (CHD) or ischaemic heart disease (IHD) [5].

Despite efforts to reduce the occurrence of CHD through healthy living campaigns and primary prevention medications, it remains the leading cause of mortality worldwide. Statistically, the worldwide mortality of CHD is predicted to rise to a staggering figure of 23.3 million by 2030 [6]. Current revascularisation therapies are mainly coronary angioplasty (followed by stenting) or coronary artery bypass grafting (CABG), aiming to open up or replace the occluded vessels. The gold standard vessel grafts with a small diameter (under 6mm) to be used in CABG surgeries are autologous, namely the saphenous vein or internal thoracic artery. The internal thoracic artery is the most effective conduit, with patency rates between 85–95% over 7 to 10 years [7,8]. The saphenous vein is the best with regard to ease of harvesting, but more liable to graft failure down the line [9]. However, the healthy autograft vessels used for CABG can be in short supply in some patients. It is estimated that 20–30% of patients who require a CABG surgery do not have suitable autologous vessels to be used as grafts [10]. Factors damaging vessel quality include comorbidities such as diabetes, as well as long-standing peripheral artery disease. This creates a huge demand for alternative sources of vascular conduits—namely tissue engineered vascular grafts (TEVGs) [11].

**Figure 2:**
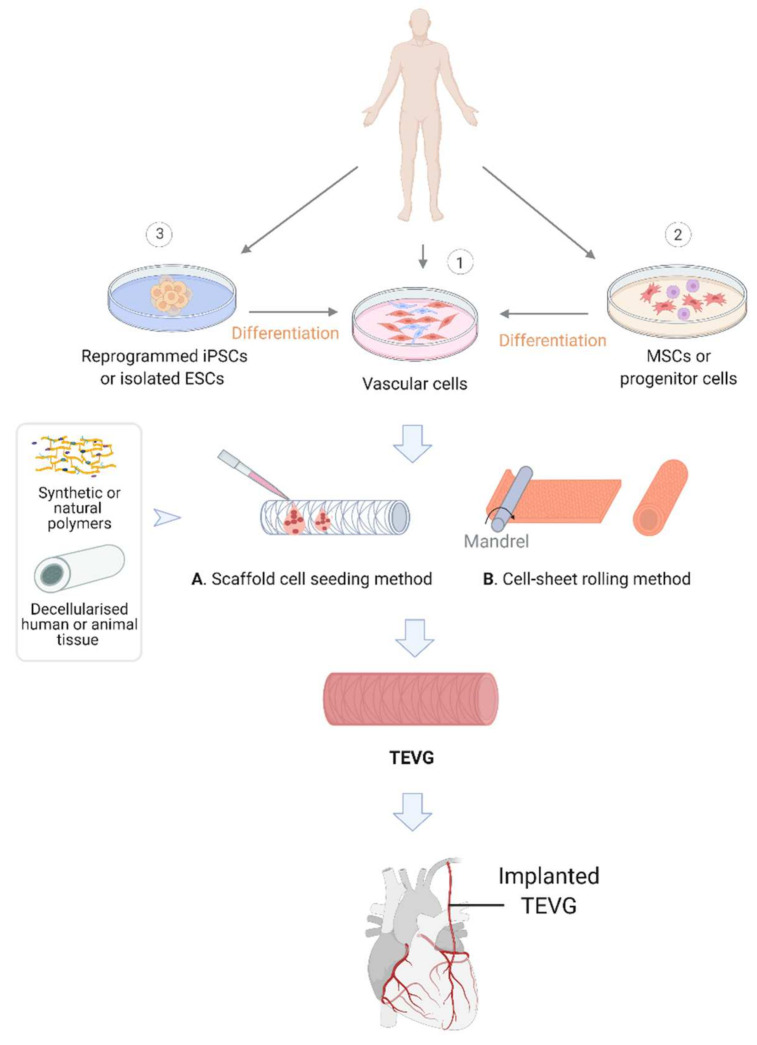
Vascular tissue engineering. Vascular cells can either be harvested from donors (1) or differentiated from mesenchymal stem cells (MSCs) or progenitor cells isolated from donors (2). Vascular cells can also be differentiated from pluripotent cells such as isolated embryonic stem cells (ESCs) or induced pluripotent stem cells (iPSCs) that were reprogrammed from somatic cells (e.g., dermal fibroblasts or blood monocytes) of the donor (3). A-Scaffold-based tissue-engineering: Vascular SMCs and ECs are seeded onto scaffold materials that can either be synthetic polymers or decellularised vascular scaffolds. B-Scaffold-free vascular engineering: TEVG produced via vascular cell bioprinting or rolling sheets of autologous vascular cells into a tubular structure. The constructs from A or B are then cultured ideally in a bioreactor to develop suitable properties of a TEVG for clinical implantation such as coronary artery bypass grafting. (Created with Biorender.com, accessed on 8 January 2022).

Although engineered vascular grafts have shown promising long-term outcomes when replacing large- and medium-sized arteries, there have been poor patency rates associated with small-diameter vessels such as coronary arteries [10]. Hence there is a clear need to improve the quality and biocompatibility of small diameter vascular conduits to meet the clinical demand and provide alternative grafts for patients. Fortunately, recent technological advances have provided the possibly of producing long lasting grafts to address the clinical need. This has been achieved via different manufacturing methods. These include scaffold-free methods which use cells to produce their own matrix, or cell sheets that can then be assembled into conduits using a mandrel or rod, as well as more traditional methods that use polymer based or decellularised tissue scaffolds that can be laden with cells to fabricate vessel-like conduits (Figure 2) [12,13,14,15]. This report will explore the advancements and challenges associated with vascular engineering, with a focus on small diameter vessels.

## 2. Design Requirements for TEVGs

**Figure 3:**
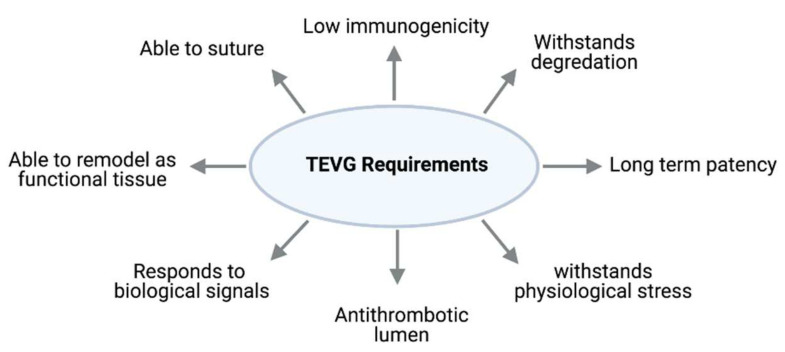
Key factors to be considered for an ideal TEVG.

For a successful TEVG, it is important to identify the key design requirements [16]. Since the grafts are to be implanted to support blood flow in vivo, certain mechanical properties are required to prevent leakage, rupture or aneurysm formation [17]. Firstly, the graft should have adequate burst pressure and compliance properties that can handle the physiological blood flow at the implantation site. Secondly, the grafts must be biocompatible to the host tissue and survive in the in vivo environment by having minimum immunogenicities to minimise inflammation and avoid rejection. Moreover, grafts are required to integrate with the anastomosing vessels in vivo and promote angiogenesis at the implantation site [18]. This means they require the capability of self-repairing and remodeling to avoid graft failure and provide longevity. Specific to small diameter vessels, the lumen must possess an endothelial layer to prevent thrombus formation [19]. Figure 3 summarised key factors to be considered when choosing the materials, cell types and manufacturing processes to produce an ideal TEVG.

## 3. Scaffolds for Vascular Tissue Engineering

Functions of blood vessels are supported by the ECM that also varies in composition and architecture according to vessel types. The ECM not only provides support and anchorage for cells which are critical for cell survival, but also regulates cell behaviours such as directing cell proliferation and migration, as well as sequestering growth factors [4]. These properties are integral to the biological adaptation of TEVGs and their response to physiological signals. Hence, engineered vascular conduits are required to possess a medium that has similar functions as the ECM. One way to provide this is through a scaffold, which acts as a matrix for cells to organise into 3D structures. Therefore, an ideal scaffold should replicate the functionalities of the ECM to support the biological and mechanical properties of blood vessels for in vivo applications [20]. The scaffold should be non-thrombogenic and non-immunogenic, and have a suitable elasticity and pore size to accommodate cells growing within it. Moreover, scaffolds need to support cell growth, differentiation and tissue regeneration processes. Furthermore, an ideal scaffold is biodegradable and allows the implanted cells to integrate with the native vessels in vivo [21,22]. There are a wide variety of materials that can be used as scaffolds, ranging from natural products to synthetic materials, or a mixture of both. 

### 3.1. Natural Polymers

Natural polymers offer a potential source for scaffold materials mainly due to their biocompatibility [23]. One such popular polymer is collagen, which is the most abundant protein in the ECM and is required for both weight-bearing and supporting cell function [24]. The collagen possesses a low antigenicity and thus reduces the risk of immune responses. Collagen, among other natural polymers such as elastin and fibrin, can be extracted from animal sources such as bovine tendons or porcine skin, making it widely available and cost effective [25]. However, a drawback of these sources is the batch-to-batch variability and potential pathogenic contamination [19]. In addition, although studies have shown success in venous systems, collagen-based scaffolds could not cope well with the pressure of the arterial system [24,26]. Moreover, there are ethical considerations when harvesting tissues from animals. This has led to the exploration of synthetic polymers to obtain alternative sources of ECM proteins. 

### 3.2. Synthetic Polymers

To address the mechanical shortcomings of natural polymers, vascular engineering moved towards using biodegradable synthetic polymers for scaffolding materials. Polyglycolic acid (PGA), polylactic acid (PLA) and polycaprolactone (PCL) are three of the most commonly used synthetic polymers [27]. These synthetic polymers are advantageous for their mechanical properties that can be tailored to meet the clinical needs in terms of degradation rate, elasticity and compliance. Moreover, these materials are cheap, readily available, and free of ethical issues that would arise from using natural polymers from animals [28]. An initial study showed that vessels engineered with PGA as a scaffold yielded strong grafts with rupture strengths exceeding that of the saphenous vein [22]. However, disadvantages of these materials involve their biological performance (Table 1). Breakdown products can also induce an inflammatory response and VSMC de-differentiation [29,30]. Although surface modifications can be made to improve biocompatibility issues, the bioactivity of the breakdown products should be more thoroughly investigated to ensure long-term safety of the scaffold [31,32]. Synthetic scaffolds can be blended or conjugated with natural polymers to improve biocompatibility [33,34]. Such strategy could generate well-rounded hybrid scaffolds to achieve overall adequacy with regard to mechanical properties and biocompatibility. Nevertheless, such hybrid scaffolds could still possess some of the limitations carried over from the original materials [35,36].

### 3.3. Decellularised Scaffolds

In parallel to the synthetic methods, decellularisation of native tissues has also been trialed to generate scaffolds. Animal tissues, such as arteries, are usually used for this purpose by being decellularised using chemical agents, enzymes or physical agitation (Figure 2). This method could maximally preserve the native ECM components and help maintain the mechanical properties of the tissue [11,40]. The decellularised scaffold is then used for the seeding of autologous or other sources of vascular cells in vitro. For example, cells from the descending aorta of fetal pigs were removed by trypsin, ribonuclease and desoxyribonuclease. Porcine aortic endothelial cells were then seeded onto the scaffold and demonstrated an excellent cell viability within scaffolds [40]. A recent study has seeded human pluripotent stem cell-derived vascular progenitors onto a decellularised rat vascular scaffold. By perfusion of the cell-laden scaffold with a defined medium containing PDGF-BB or VEGF-A165 and SB431542, the vascular progenitor cells were successfully differentiated into SMCs and ECs in situ. The recellularised scaffolds were then connected to the rat circulation, which is capable of supporting peripheral blood flow in vivo [41]. Another study differentiated xeno-free ECs from human induced pluripotent stem cells, which successfully endothelialised decellularised human umbilical cord arteries in a bioreactor with a circulatory culture medium [42]. Decellularised human umbilical artery patches were also used to accommodate human adipose stem cells for vascular engineering [43]. Although the approaches of decellularisation and recellularisation could potentially simplify the production of vascular scaffolds, there have been studies which suggest that this method does not pose any clear advantages compared to the synthetic alternatives. The decellurised scaffolds are more costly than the synthetics, and have the potential transmission of pathogens or elicit immunogenic response, leading to graft failure (Table 1) [37,44]. Treatment with glutaraldehyde can reduce the immune response of recipients to the xenogeneic tissues through its cross-linking function; however, this reagent has also been shown to induce inflammatory reactions that could contribute to graft failure [45,46]. One such mechanism is through increased calcification due to the addition of free aldehyde residues to the ECM scaffold, which is correlated with increased mechanical stress [47,48].

### 3.4. Scaffold-Free Techniques

The potential complications of scaffolds on inflammation, thrombosis and rejection, and the challenges for the scaffold to mimic native ECM components for optimal cell-cell interactions and alignments have led to the emergence of scaffold-free techniques, mainly self-assembly (Figure 2) [49,50]. As pioneered by L’Heureux et al. [51], this approach involves producing sheets of autologous vascular cells, which are then shaped into a tubular structure. These are then conditioned in a bioreactor to allow the layers to fuse and produce their own ECM components [48,51]. This strategy has proved to be the first method to produce a TEVG with physiological mechanical properties without the presence of a scaffold. In a human clinical trial for haemodialysis, these grafts demonstrated a burst pressure around 2600 mmHg, well above that of the human saphenous vein [39]. It is important to note that haemodialysis applies supraphysiological flow rates to the graft, and also involves repeated needle punctures that apply additional stress on the graft. Therefore, the lifeline graft underwent extensive validation before the application [52]. Results from this study were promising, with seven out of nine patients (78%) maintaining primary patency one month after implantation. Moreover, five out of remaining eight patients (60%) maintained primary patency after six months. These results approach the objectives of the Dialysis Outcomes Quality Initiative of 76% three months after implantation for native vein fistulas. The main reasons for graft failure in this study were thrombosis and aneurysm formation. On the downside, these grafts are expensive and have a long production time ranging from six to nine months [51]. However, recent studies by Jung et al. [50] and Saito et al. [53] have shown the feasibility of reducing production time to as low as 35 to 48 days using stem cells or progenitor cells [50,53].

## 4. Cell Sources Used for Vascular Tissue Engineering

### 4.1. Autologous Vascular Cells

ECs and VSMCs are of paramount importance to the structure and function of blood vessels, and thus equally important for TEVGs. A primary source of ECs and VSMCs is from the patients themselves, and these are also known as autologous vascular cells [16]. Although the advantage of this source is immune compatible, there are a number of drawbacks. Firstly, these cells are usually harvested through blood vessel biopsies, which is invasive and has a risk of donor site complications. Secondly there are limitations regarding the quantity of obtainable cells that usually have poor proliferative and regenerative capacities due to the advanced age of donors and the primary nature of the cells. Although the issue concerning proliferation was addressed through the expression of a human telomerase reverse transcriptase subunit, the age-associated senescence remains a challenge [54]. It is important to note that the use of genetic manipulation requires long-term follow-ups in vivo before being applied clinically. Furthermore, the process of harvesting and culturing primary cells is expensive and time consuming, thus presenting financial barriers. The challenges associated with using primary cells from patients led to efforts of acquiring ECs and VSMCs from stem cell-based approaches.

### 4.2. Embryonic Stem Cells

**Figure 4:**
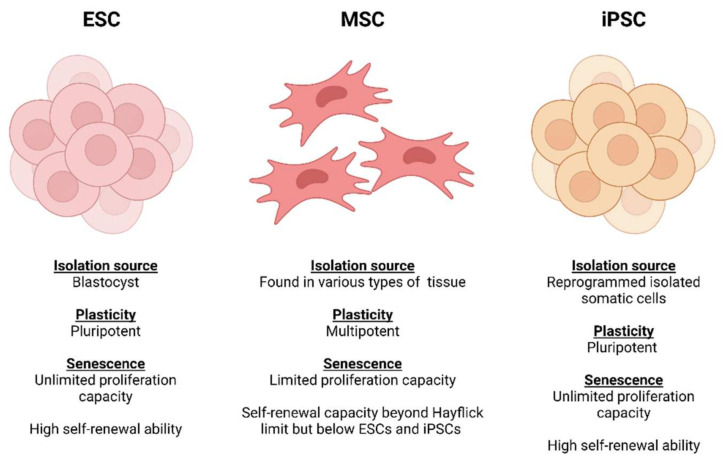
Comparisons of plasticity potential, source of extraction and senescence between stem cell sources (Created with Biorender.com, accessed on 8 January 2022).

In recent years, stem cells have emerged as a promising source for vascular engineering. The major advantage of stem cells is their ability of self-renewal and differentiation in accordance with the conditions applied. Broadly speaking, these come in two main categories: embryonic and adult stem cells (Figure 4) [55,56].

Embryonic stem cells (ESCs) are derived from embryos at the blastocyst stage and possess the ability to differentiate into cells from all three germ layers (ectoderm, mesoderm and endoderm) [57]. These were shown to be effective in an in vivo mouse study, where human ESC-derived ECs were transplanted into mice and able to integrate into the host blood vessels and served as a vascular conduit that was functional for 150 days [58]. However, this has not yet been attempted in human studies. One of the main reasons is due to the risk of teratoma formation, which raises serious safety concerns [58,59]. Moreover, there is an ethical dilemma surrounding the use of human ESCs [60].

### 4.3. Mesenchymal Stem Cells

Mesenchymal stem cells (MSCs) are cells with multipotent plasticity and self-renewal ability with mesodermal lineage differentiation potential [61]. The first successful isolation of MSCs was from mice bone marrow, reported by Friedenstein and colleagues in 1966. This was later achieved in humans by Haynesworth in 1992 [62,63]. Since then, MSCs have been discovered to reside in various types of adult and fetal tissues as well as being obtainable from multiple sources including dental pulp, tendon, muscle, umbilical cord, skin, liver, peripheral blood, hair follicle and adipose tissue. Due to their broad distribution and unique biological properties, MSCs have been extensively studied over last three decades for tissue engineering and regenerative medicine applications [64,65,66].

With the capability of differentiating into different lineages of mesodermal origin, MSCs have myogenic differentiation potential. Therefore, MSCs have been used to derive SMCs via the introduction of defined chemical factors and mechanotransduction signals. MSC derived VSMCs have been used in various blood vessel tissue engineering strategies to mimic the tunica media of the native vasculature [67,68,69]. Gong et al. [70] reported the first use of MSCs for engineering small-diameter blood vessel mimics. Using bone-marrow derived MSCs laden on tubular PGA mesh scaffolds and subsequent seeding of ECs to endothelialise the luminal surface, Gong et al. [70] fabricated a vessel mimic with MSC derived VSMCs that expressed SMC-specific markers and secreted native ECM proteins. The engineered vessels displayed a similar morphology to those of native vasculature [70]. More recently, Lacobazzi et al. [71] fabricated TEVGs by seeding the surface of a CorMatrix decellularised commercial cardiac patch with thymus derived MSCs. The TEVGs grafted on piglet left pulmonary artery models remained patent with no evidence of stenosis, rupture, thrombosis or tissue degradation three months post-engraftment. Furthermore, an organized VSMC population, an endothelialized luminal surface and a vascularised outer layer could be observed on the explanted TEVGs [71].

Compared to ESCs, MSCs have a more limited multipotent plasticity and rapidly lose their differentiation potency and telomerase activity during in vitro expansion due to senescence [72,73]. Furthermore, acquiring an acceptable quantity of MSCs from a single source has been identified as a major issue which hinders MSCs for clinical use and tissue engineering [74,75]. Despite these limitations, MSCs have been reported to hold remarkable genomic stability and tend to pose less significant ethical considerations compared to ESCs [61,76]. Also, MSCs have an advantage in terms of immunosuppressive characteristics. The expression of major histocompatibility complex (MHC) class I, Toll-like receptors (TLRs) and programmed death ligand 1 (PD-L1) proteins can protect MSCs from natural killer cells (NKs). Furthermore, MSCs have been shown to secrete anti-inflammatory factors such as transforming growth factor β (TGF-β) and hepatocyte growth factor (HGF) to inhibit the activation/functions of immune cells [77,78,79]. Due to these propitious characteristics, MSCs are believed to be capable of preventing immunogenic responses in the host tissue. The safety profile of MSCs has been well documented both in vitro and in vivo to support their safety for clinical uses. This has led to the registration of over 950 MSC-based clinical trials in the last 25 years, including approximately 70 registered cardiovascular injury repair therapies in the last decade. As a result, using MSCs to fabricate TEVGs for clinical applications continues to be extensively investigated and documented. Nevertheless, there is still no MSC-derived TEVG that has reached clinical application from trials [66,74,80,81,82].

### 4.4. Progenitor Cells

Progenitor cells are another type of adult stem cell that specifically matures into its destined cell type. These progenitor cells can also be isolated from the bone marrow or blood, thus reducing the need for harvesting native vessels [83]. An ovine study showed that endothelial progenitor cell-based grafts provided effective patency, which was largely due to the production of nitrous oxide (NO) [84] that inhibits both platelet aggregation and VSMC proliferation and dilates blood vessels [85]. However, progenitor cells may be depleted in the elderly population, hence limiting their supply [86].

Adipose tissue also contains stem cells (ASCs), which can differentiate into both ECs and VSMCs [87,88]. The benefit of using ASCs is their wider availability and ease of harvest, even in the elderly population. Moreover, the number of ASCs seems unaffected by age, with evidence suggesting that their availability even increases with advanced age [89].

### 4.5. Emergence of Induced Pluripotent Stem Cells (iPSCs)

Despite the advances in employing ESCs and adult stem cells for vascular engineering, challenges including ethical issues, cell accessibility and heterogeneities remain which hinder the production of TEVGs. In 2006, there was a major breakthrough in stem cell biology—the emergence of induced pluripotent stem cells (iPSCs), which provides great potential to generate patient-specific cell types for vascular engineering [90,91,92]. Discovered by Dr Yamanaka and his team, iPSCs can be obtained via reprogramming adult somatic cells by inducing the four pluripotency factors: OCT-3/4, Sox2, Klf4 and c-Myc. The reprogrammed iPSCs possess the ability to differentiate into potentially any cell type of the three germ layers under defined conditions [90,93].

There are numerous advantages associated with the use of iPSCs in vascular engineering. IPSCs provide a potentially unlimited cell source, since they could be derived from a number of easy-access tissues of the donor including the skin or peripheral blood, in addition to their excellent self-renewal capacity [94,95]. Using autologous cells that are derived from the same patient also addresses ethical dilemmas and reduces the risk of immunological reactions caused by the allogeneic cells [94]. Moreover, vascular abnormalities caused by genetic mutations can be addressed by gene editing of the iPSCs from the patients to correct the DNA variants, which have potential therapeutic values [96,97]. A study by Luo et al. [98] demonstrated that human iPSC-derived TEVGs yielded impressive mechanical and contractile function, as well as excellent patency when implanted into a rat aortic model [98].

Although iPSCs provide a promising cell source for vascular tissue engineering, the technology also comes with a number of challenges. The most serious of these is the tumorigenesis risk associated with the pluripotent cells [99]. A study by Galat et al. [100] reported spontaneous transgene activation in iPSC vascular derivatives [100]. There were also reports that derivatives of iPSCs may not be immunologically inert due to the accumulation of mitochondrial DNA mutations. The reprogramming process can also lead to chromosomal rearrangement, thus increasing tumorigenic potential [100,101,102]. These issues present significant obstacles for the clinical use of iPSCs, as more stringent quality controls and screening measures are required, which inevitably increases the economic burden associated with iPSC therapies. Costs associated with preparing a biologically safe cell line can reach up to a million US dollars, which makes it less feasible to simply translate the technology to clinical practice [103]. Another issue is the time-consuming nature of the iPSC production, which needs to be optimised and standardised. Therefore, there is an urgent need to increase the safety, reduce the costs and improve the manufacturing processes associated with iPSCs before wider clinical applications are pursued. Table 2 highlights the advantages and disadvantages of the main cell types used in the production of TEVGs.

## 5. Cell-Seeding Techniques in Vascular Tissue Engineering

SMCs and ECs are the two major cell types to be accommodated in vascular grafts. The vascular cells come from a variety of sources, as described below. Different techniques are also employed to assist with cell seeding and growth in the scaffolds.

### 5.1. Passive Seeding

**Figure 5:**
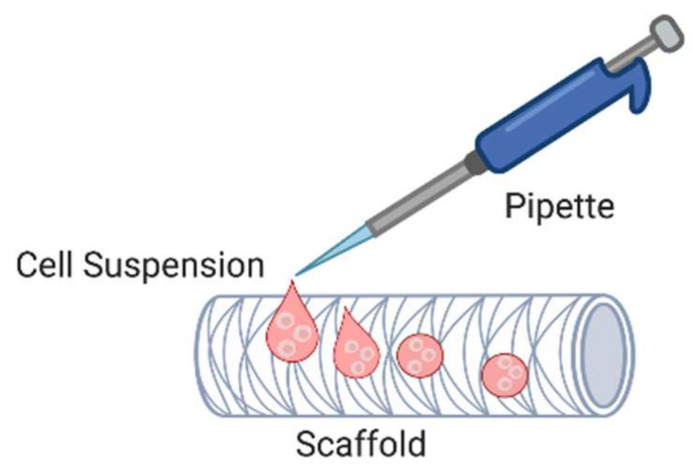
Passive seeding. Cell suspension is pipetted directly onto the lumen or exterior of the scaffold. (Created with BioRender.com, accessed on 8 January 2022).

The first reported TEVG fabrication by Weinberg and Bell in 1986 used a passive seeding method. Decades later, it remains the most common cell-seeding method used to fabricate TEVGs due to its low costs and ease of operation. Because of its simplicity, this approach is able to avoid cell damage from mechanical forces such as shear stress caused by the extensive manipulation of cells [107,108,109]. In fact, the most successful and longest ongoing TEVG clinical studies by Shin’oka and colleagues (NCT01034007 and NCT04467671) have used passive seeding to fabricate their vascular grafts [110,111,112]. This technique is performed via pipetting a cell suspension directly onto either the scaffold lumen or exterior followed by a short incubation to allow cell attachment (Figure 5) before the culture medium is added. The cell-laden scaffolds are then incubated for a period of time, and a tissue-like biological construct can be produced [109,113].

The success of cell seeding is usually evaluated by the seeding efficiency which measures the percentage of total seeded cells attaching to the scaffolds over a short period of culture (<24 h) before cell doubling. Although it is the simplest and least expensive seeding technique, passive seeding has low cell-seeding efficiency [108,114,115]. Earlier efforts resulted in seeding efficiencies approximately 25% [116]. By including a degassing step to remove air pockets in the matrices prior to pipetting the cell suspension on to scaffolds, Vitacolonna et al. [117] demonstrated that the seeding efficiency could be improved to as high as 42% [117]. Nevertheless, despite this improvement, the efficiency of passive seeding remains significantly lower compared to other techniques like dynamic or electrostatic seeding (Table 3) [114,116,118,119]. Another drawback associated with the passive seeding technique is the suboptimal and heterogenous distribution of cells across the scaffold during seeding. This can lead to poor cell infiltration which inapt the recellularisation of scaffolds in the host and hinders ECs or mural cells from aligning similarly to the native vessels due to haphazard adhesion of the cells on the scaffold and the lack of physiological mechanotransduction cues during culture [120,121,122].

To enhance the efficiency of the passive seeding, coating strategies have been used to overlay the surface of scaffolds with bioactive components. Biomaterials that are constituted of common ECM components found in the native vasculature such as fibronectin, fibrin as well as collagen or other natural sources like silk fibroin, have been used to coat the scaffold surfaces, and this has been shown to improve cell attachment and retention [137,138,139,140]. Nevertheless, despite these efforts, thrombogenicity remains prominent in TEVGs fabricated via passive seedling [137,141]. By conjugating coatings with anticoagulants or administering anticoagulant drug therapies, studies have attempted to reduce the rates of thrombosis. However, these alternatives give rise to other complications for patients requiring revascularisation surgery or other surgical procedures, as coagulation mechanisms are pivotal in wound healing, and therefore their inhibition increases the risk of haemorrhage [110,142,143]. These limitations have led to investigating alternative seeding techniques to improve seeding efficiency and the performance of TEVGs.

### 5.2. Dynamic Seeding

**Figure 6:**
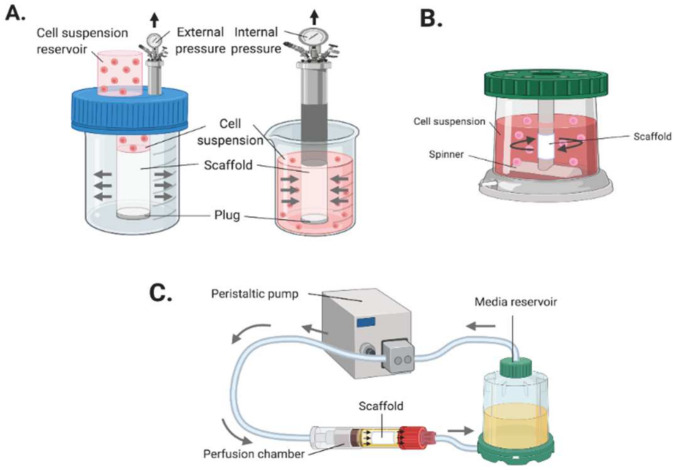
Dynamic seeding methods. (**A**) Vacuum seeding: using internal or external pressure forces to drive cells into scaffolds. (**B**) Centrifugal/rotational seeding: using rotational force to drives cells into the scaffold. (**C**) Perfusion seeding: mimicking the in vivo physiological conditions and biomechanical stress of blood vessels to aid cell attachment to the scaffold. (Created with BioRender.com, accessed on 8 January 2022).

Dynamic seeding is a method that uses external forces during the seeding of cells onto a substrate of scaffold. This method has been shown to yield a more uniform or homogeneous distribution and penetration of adherent cells across seeded scaffolds compared to other methods [127,130,144]. Dynamic seeding can be applied via a wide range of techniques (Figure 6) using pressure, perfusion, stress, strain or centrifugal/centripetal force systems [145,146,147,148]. A common technique, vacuum seeding, essentially forces the cell suspension through the micropores of the engineered graft by utilising a pressure differential system [149]. Other typical techniques like perfusion or centrifugal systems use bioreactors to exert similar fluid dynamic forces as those observed in vivo or by driving cells onto a substrate via hydrostatic pressure from rotating inertial forces [111,130,150,151]. These methods present a rapid solution to achieving excellent seeding efficiencies of up to ~90% [108,149]. Furthermore, these systems are cheap and disposable and can be configured as an automated process to enhance reproducibility for clinical applications [152]. However, each technique has its own drawbacks. Reduced cell viability and changes in gene expression or cell morphology has been associated with centrifugal/vacuum methods. The complexity of the bioreactor systems and the long culture period are common disadvantages attributed to the perfusion techniques [113,130,148]. Complications such as failure, fatigue, and contamination are prominent risks with the prolonged culture of cells in bioreactors [108].

### 5.3. Electrostatic Seeding

**Figure 7:**
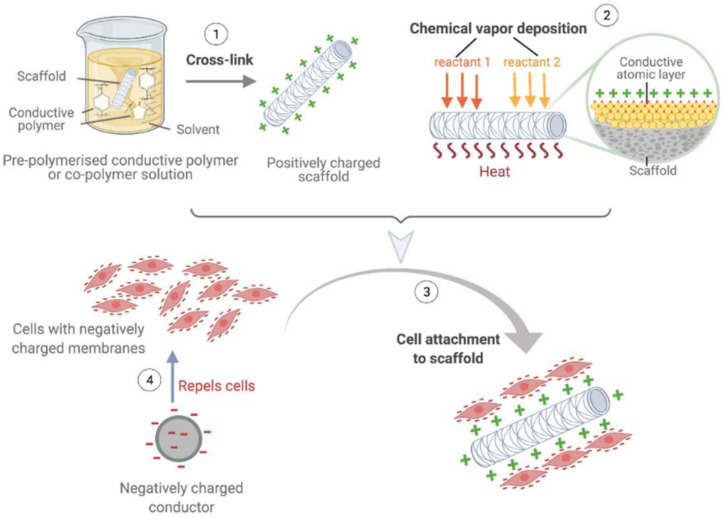
Electrostatic seeding. Scaffolds are manipulated to become positively charged substrates to attract the negatively charged regions on cell membranes for increased retention and attachment of cells. Scaffolds can be chemically modified by either cross-linking polymers in pre-polymerised solutions during the fabrication process (1), or by covering scaffold surfaces with a thin conductive layer via atomic layer deposition post-fabrication (2). The negatively charged cells can then be seeded on to the positively charged scaffold (3). Negatively charged conductors can also be employed to repel cells to increase the efficiency of cell attachment on scaffolds. (Created with BioRender.com, accessed on 8 January 2022).

The structure of the plasma membrane in mammalian cells is arranged in a phospholipid bilayer with negatively charged hydrophilic and non-polar hydrophobic regions [153,154]. Phosphates present in the hydrophilic heads of membranes provide the external surface with a negative charge. Electrostatic cell seeding techniques utilize the negatively charged characteristics of cell membranes by manipulating the electrostatic properties of the scaffold to promote cell attachment (Figure 7) [155,156,157]. This technique can yield seeding efficiencies as high as ~90%. Furthermore, electrostatic seeding has been shown to accelerate the maturation of cells via electrostatic phospholipid interactions and improve cell retention post-implantation, which decreases the risk of graft failure [104,134,135,136]. While this technique shows promise, it also has limitations. Via electrostatic forces, cells are driven to adhere to the surface of substrates; therefore, cells cannot be deeply embedded within scaffolds to generate tunica media-like structures. Additionally, high electrical conductivity in substrates may interfere with proper focal adhesion complex formation, which could reduce the proliferation potential of seeded cells [105]. Furthermore, there is a lack of studies regarding the long-term effects on cell viability and overall biological functions of TEVGs produced via electrostatic seeding methods. Hence, further research on its long-term outcomes in vivo is still required before translating this method to clinical use [105,108,149]. 

## 6. Maturation of TEVGs

Once a TEVG has been loaded with cells, the next step is ensuring that it develops and matures into an effective graft for the implantation in vivo. As previously mentioned, there are mechanical requirements that need to be achieved to warrant the implantation of a TEVG. To ensure these criteria are met, the grafts need to go through a conditioning process for maturation and require proper testing prior to implantation [158]. This can be achieved through the introduction of pulsatile flow to remodel the vessel. Niklason et al. [159] proved that this process is necessary for VSMC migration throughout the scaffold [159]. Moreover, this mechanical stress leads to VSMC proliferation and differentiation, as well as ECM remodeling [98,159].

Following these findings, Niklason and colleagues pioneered the development of bioreactors as in vitro biomimetic flow systems [159]. By emulating the physiological conditions that blood vessels experience in vivo, other groups also demonstrated that the TEVGs matured to mimic the properties of native vessels through increased ECM formation, VSMC and EC differentiation, as well as migration [160,161]. Advancements in bioreactor technology have also minimised the risk of contamination through the automation of tissue culture, wireless data transfer and pH monitoring. However, the complexity of conditions required to produce grafts that fit these purposes remains a challenge [160,162].

A major limitation of vascular conduits is the post implant stenosis secondary to excessive SMC proliferation, which is also known as intimal hyperplasia. This can lead to the loss of contractility of the vessel, which ultimately results in graft failure [163]. VSMCs express a spectrum of contractile and proliferative markers [164]. A high proliferative index is associated with a higher teratogenic potential, and vice versa. The characteristics of the contractile phenotype of VSMCs include the sensitivity to small molecular signalling (such as acetylcholine and noradrenaline), a high expression of contractile apparatus proteins, and a low proliferative index. On the other hand, the proliferative phenotype exhibits extensive ECM synthesis, low expression of contractile proteins, and a high proliferative index [163]. Studies have shown that it is possible to manipulate VSMCs to promote the expression of contractile marker genes to improve functioning. This can be achieved through signalling molecules such heparin and TGF-β1, which inhibit proliferation and induce the expression of contractile proteins such as α-SMA [165]. Additionally, studies have indicated that cyclic strain could increase the expression of the contractile genes as well as stimulate the production of ECM components which are necessary for cell survival [163]. Therefore, this provides further insight into the conditioning of seeded cells, which can aid the development of TEVGs.

## 7. Clinical Applications

Vascular tissue engineering has existed since the 1950s, but it was Weinberg and Bell who produced the first TEVG consisting of the main three layers (intima, media, adventitia) in 1986 [107]. Their approach used a combination of xenogeneic bovine vascular cells and collagen gels. Although this graft was weak (yielding a burst pressure of 10 mmHg), it gave vascular engineering a concept to build on and improve. Since then, several TEVGs have reached the clinical setting (Table 4).

The first reported clinical application of a TEVG was performed by Shin’oka’s group in 2001. This was a biodegradable pulmonary conduit composed of peripheral vein derived primary VSMCs from the same individual and PGA reinforced scaffold which was implanted in a child with congenital pulmonary atresia [174]. Results were positive, with patency being maintained for seven months post implantation. This study was then expanded to a further 23 paediatric patients with the same condition (NCT01034007) [166]. Follow-up at 5.8 years showed no graft-related mortality [110,167]. More recently, a second-generation TEVG derived from bone marrow mononuclear cells seeded on PGA and PCLA (Polycaprolactone-co-lactide) copolymer scaffolds by Shin’oka’s group has been approved for a clinical trial in the safety phase (NCT04467671) This project has been active since July 2020 and is looking to improve on the outcomes of its predecessor [82,175].

L’Heureux subsequently developed the first sheet-based TEVG, the Lifeline graft. This approach utilises the induced production of ECM through culturing and maturation of autologous cells, rather than using an exogenous scaffold [38]. The graft was first tested in animal models, which confirmed that its mechanical stability was considerably higher than that of the human saphenous vein. Moreover, there was a good integration of the graft with the surrounding tissue, and no thrombus formation [52]. Following these promising results, the Lifeline graft was implanted into nine patients with end stage renal disease as part of a clinical trial (NCT00850252). Out of these, six patient had patent grafts at six months, whereas the other grafts failed from thrombosis or rejection [39]. Mechanical testing was undertaken before implantation, which confirmed that the average burst pressure was 3490 mmHg, matching the human internal mammary artery. However, the main drawback of the graft was the production time, which ranged from six to nine months, which is far too long and expensive for it to be introduced into routine clinical practice [52].

Since then, there has been a shift towards the use of decellularised scaffolds for engineered vessels—a key player being the Humacyte graft. Lawson et al. [170] used the grafts in clinical trials for end stage renal failure patients to provide AV shunt access for haemodialysis (NCT01744418 and NCT01840956) [169,170,171]. Results showed 63% patency at six months, but only 18% at 18 months. These results still need to be improved in order to justify the high cost of the production. However, more recently, the Humacyte grafts have entered a new clinical trial (NCT03005418) to treat damaged vessels that require repair after vascular trauma, and they are currently recruiting for Phase 2 clinical trials [173]. Nonetheless, the results for their AV shunt application still need to be improved to justify the high cost of the production. Going forward, the focus needs to be on simplifying the design and manufacturing processes for TEVGs in order to produce off-the-shelf grafts for patients in urgent need of the product. Although Dahl et al. [176] laid the foundations for this concept, there is still a long way to go to validate a product for routine clinical practice.

## 8. Conclusions and Future Perspectives

The ultimate goal of vascular engineering is to produce clinically effective small-diameter conduits that could integrate successfully in vivo to replace diseased vessels and meet the high demand for surgeries such as CABG [10]. The engineered vessel grafts need to resist thrombosis, maintain patency and withstand physiological stresses. Despite best efforts, the engineered vascular grafts still have not matched the performance of autologous vessels, therefore, there have not been commercially available small-diameter grafts [19]. More research needs to be undertaken to confirm the optimal cell types, scaffolding techniques and conditioning requirements to develop clinically appropriate TEVGs. Efforts should also be made to further our understanding of the in vivo integration and remodeling of small-diameter grafts to facilitate the innovation. Moreover, technologies for producing ‘off-the-shelf’ grafts which would ensure the availability of TEVGs in various clinical scenarios are waiting to be developed. It is likely that a combination of clinical outcomes and economic considerations will dictate the approaches and materials to be utilised for wider clinical applications.

## Figures and Tables

**Table 1:** Advantages and disadvantages of different scaffold techniques in the production of TEVGs.

Vessel Type	Advantages	Disadvantages	References
Natural Scaffolds	Lack of immunogenicity Cheaper & readily available source	Weaker mechanical properties Risk of pathogenic contamination Batch to batch variability	[23,24,25]
Synthetic Scaffolds	Highly reproducible Tailorable mechanical properties	Risk of immune rejection and thrombogenicity	[28,29,30]
Decellularised Matrix	Preservation of ECM components and mechanical architecture	More expensive Risk of immune rejection if not completely decellularised	[11,37]
Self-assembled cell sheets	Mimics native ECM components Lack of immunogenicity	Long fabrication time Mechanical properties vary according to cell type	[38,39]

**Table 2:** Advantages and disadvantages of different cell types in vascular engineering.

Cell Category	Cell Type	Advantages	Disadvantages	Reference
Autologous somatic cells	Vascular derived ECs, SMCs and fibroblasts, Dermal fibroblasts	Easy sourcing, culture and expansion. Immune compatibility.	Invasive harvesting risks donor site complications. Limited proliferative and regenerative capacities.	[16,51,104]
Progenitor cells	Vascular endothelial progenitor cells, Bone marrow-derived SMC progenitor cells	Able to isolate from bone marrow and blood. Greater replicative and regenerative potential.	Availability may be depleted in the elderly population.	[80,83,106]
Mesenchymal stem cells	MSC derived SMCs	May be extracted from multiple sources. Remarkable genomic stability.	Limited differentiation into ECs. Rapidly lose their differentiation potency during in vitro expansion.	[62,64,70,71,74,75,76,77]
Embryonic stem cells	ESCs	Ability of self-renewal. Can be differentiated into vascular ECs and SMC.	Safety concerns (risk of teratoma formation). Ethical issues.	[57,58]
Human induced pluripotent stem cell (hiPSC)	Wide range of somatic cells	Can be differentiated into any cell type. Excellent self-renewal capacity. Reduced risk of immunogenicity.	Risk of tumorigenesis. Expensive process.Time consuming process.	[90,92,96,100]

**Table 3:** Comparison of cell seeding efficiencies * between passive, dynamic (centrifugal, vacuum and perfusion) and electrostatic cell seeding methods.

Cell Seeding Method	Seeding Efficiency	Reference
Passive seeding	10–42%	[110,116,117]
Centrifugal Seeding	~40–90%	[116,123,124,125]
Vacuum Seeding	90%≥	[109,126,127,128]
Perfusion Seeding	50–90%	[129,130,131,132]
Electrostatic Seeding	~90%	[133,134,135,136]

**Table 4:** TEVGs that have reached clinical trials.

Identifier	Application	Cell Type	Scaffold	Status	Reference
NCT01034007	Cavopulmonary shunt	Primary VSMCs	PGA	Completed	[106,166]
NCT04467671	Cavopulmonary shunt	Bone marrow mononuclear cells	PGA and PCLA	Recruiting	[82,167]
NCT00850252	AV shunt	Primary ECs and Fibroblasts	Scaffold-free	Completed	[39,168]
NCT01744418	AV shunt	Decellularised VSMCs	PGA	Active, not recruiting	[169,170]
NCT01840956	AV shunt	Decellularised VSMCs	PGA	Completed	[170,171]
NCT03005418	Vascular injury repair graft	Decellularised VSMCs	PGA	Recruiting	[172,173]

## Data Availability

Not applicable.
